# 740 Cyclosporine in Stevens Johnson Syndrome & Toxic Epidermal Necrolysis: Experience from a Tertiary Care Center

**DOI:** 10.1093/jbcr/irac012.293

**Published:** 2022-03-23

**Authors:** Manal Hassan, Bao Vincent Ho, Dhaval Bhavsar, Richard Korentager

**Affiliations:** University of Kansas Medical Center, Kansas City, Kansas; University of Kansas School of Medicine, Kansas City, Kansas; University of Kansas Health System, Kansas City, Kansas; University of Kansas Medical Center, Kansas City, Kansas

## Abstract

**Introduction:**

Stevens-Johnson syndrome and toxic epidermal necrolysis (SJS/TEN) are rare severe cutaneous adverse reactions associated with high morbidity and mortality, however, there lacks an established treatment protocol. Treatment with intravenous immunoglobulin (IVIg) has demonstrated mixed success rates in improving mortality. More recently, cyclosporine (CsA) has been found to have a promising role in management of SJS/TEN owing to its potent anti-apoptotic activity. We present 3 patients with SJS/TEN treated in our burn unit with CsA; all of them demonstrated clinical improvement.

**Methods:**

We conducted a retrospective analysis of patients who were hospitalized with the diagnosis of SJS/TEN in a specialized burn center in the year 2020. Data regarding clinical factors, causative agent(s), disease severity, treatment received, and outcome were collected on chart review. SCORTEN and ABCD-10 prognostic scores were calculated for each patient at the time of admission. All patients were evaluated by a dermatologist and started on CsA at a dosage of 5 mg/kg daily given in 2 divided doses. Predicted mortality rate was compared with observed mortality rate to assess treatment outcomes.

**Results:**

A total of 3 patients were identified with a mean age of 48.33 ± 16.50. All patients had TEN with an average initial BSA involvement of 56.67 ± 20.21 and peak BSA involvement of 63.33 ± 25.17. Lamotrigine was the presumptive causative drug in each case with an average duration of time from starting the medication to symptom onset of 19.3 days. Patients received CsA on average 7 days after symptom onset. Most of our patients had oral (100%), ocular (100%), genital (100%), and esophageal (33.3%) involvement during hospitalization. The observed mortality rate of 0% was lower than that predicted by SCORTEN (12.2% to 32.4%) and ABCD-10 (5.4% to 12.3%). No patients developed complications during admission. All patients had minimal wound care needs on discharge as most of the involved areas were healed by then.

**Conclusions:**

Our study sheds light on a possible beneficial role of cyclosporine for the treatment of SJS/TEN and reinforces the necessity of further prospective studies to solidify treatment guidelines.

